# A natural variation in *SlSCaBP8* promoter contributes to the loss of saline–alkaline tolerance during tomato improvement

**DOI:** 10.1093/hr/uhae055

**Published:** 2024-02-23

**Authors:** Jian Liu, Chi Zhang, Heyao Sun, Yinqiang Zang, Xianwen Meng, Huawei Zhai, Qian Chen, Chuanyou Li

**Affiliations:** College of Agronomy, Shandong Agricultural University, Tai’an, Shandong 271018, China; College of Agronomy, Shandong Agricultural University, Tai’an, Shandong 271018, China; College of Agronomy, Shandong Agricultural University, Tai’an, Shandong 271018, China; College of Horticulture Science and Engineering, Shandong Agricultural University, Tai’an, Shandong 271018, China; College of Horticulture Science and Engineering, Shandong Agricultural University, Tai’an, Shandong 271018, China; College of Horticulture Science and Engineering, Shandong Agricultural University, Tai’an, Shandong 271018, China; Beijing Key Laboratory for Agricultural Applications and New Techniques, Plant Science and Technology College, Beijing University of Agriculture, Beijing, 102206, China; College of Life Science, Shandong Agricultural University, Tai’an, Shandong 271018, China

## Abstract

Saline–alkaline stress is a worldwide problem that threatens the growth and yield of crops. However, how crops adapt to saline–alkaline stress remains less studied. Here we show that saline–alkaline tolerance was compromised during tomato domestication and improvement, and a natural variation in the promoter of *SlSCaBP8*, an EF-hand Ca^2+^ binding protein, contributed to the loss of saline–alkaline tolerance during tomato improvement. The biochemical and genetic data showed that *SlSCaBP8* is a positive regulator of saline–alkaline tolerance in tomato. The introgression line Pi-75, derived from a cross between wild *Solanum pimpinellifolium* LA1589 and cultivar E6203, containing the *SlSCaBP8*^*LA1589*^ locus, showed stronger saline–alkaline tolerance than E6203. Pi-75 and LA1589 also showed enhanced saline–alkaline-induced *SlSCaBP8* expression than that of E6203. By sequence analysis, a natural variation was found in the promoter of *SlSCaBP8* and the accessions with the wild haplotype showed enhanced saline–alkaline tolerance compared with the cultivar haplotype. Our studies clarify the mechanism of saline–alkaline tolerance conferred by *SlSCaBP8* and provide an important natural variation in the promoter of *SlSCaBP8* for tomato breeding.

## Introduction

Saline–alkaline soil is a major abiotic stress affecting crop productivity and quality in the world [1, 2]. Over 950 million hectares of soils are affected by saline–alkaline stress in the world [3]. Saline–alkaline soil with high levels of sodium bicarbonate and sodium carbonate is characterized by high salt and high pH (above pH 8.0) [4]. Relative to the neutral salts, saline–alkaline salts damage plants much more severely due to the combination of high pH stress, secondary stresses, ion toxicity, and osmotic stress [5–7].

To defend against saline–alkaline stress, plants have developed adaptive strategies. Under saline–alkaline conditions, Ca^2+^ binding ZmNSA1 (Na^+^ content under saline–alkaline condition) increases the transcription of plasma memrane-H^+^-ATPases (*MHA*s) and promotes Na^+^ efflux mediated by the Na^+^/H^+^ antiporter SOS1 in maize [8]. The Gγ subunit AT1 (Alkaline Tolerance 1) negatively modulates the phosphorylation of PIP2 aquaporins and reduces the H_2_O_2_ export activity of PIP2s, leading to the over-accumulation of H_2_O_2_ and resulting in alkaline stress sensitivity in sorghum, millet, rice, maize, and wheat [9, 10]. SCaBP3/CBL7 decodes alkaline-mediated Ca^2+^ signaling and releases SCaBP3 inhibition on activities of PM-H^+^-ATPase AHA2 against alkali stress in *Arabidopsis* [11].

To deal with ion toxicity, plants activate the Salt-Overly-Sensitive (SOS) pathway. Salt stress promotes calcium accumulation in the cytoplasm. The Ca^2+^ signaling is decoded by two EF-hand calcium-binding proteins, SOS3 in roots and SCaBP8 in shoots. SOS3 and SCaBP8 bind and activate the serine/threonine protein kinase SOS2. Then SOS2 phosphorylates and activates SOS1 to extrude the excess Na^+^ from the cytosol to the apoplast [12]. To prevent over-activation of SOS signaling, the SCaBP8–SOS2 complex represses the activity of a putative calcium-permeable transporter, AtANN4, by directly interacting and phosphorylating AtANN4, which fine-tunes AtANN4-dependent calcium transients under salt stress [13].

The ancestors of modern crops provide resourceful natural variations for revealing the mechanism of salt tolerance [2, 14, 15]. *OsHKT1;1* regulates root Na^+^ content and *OsHKT1;1* from indica uptakes Na^+^ with much more efficiency than *that from japonica*, which contributes to the tolerance difference between *indica* and *japonica* in rice [16]. Genome-wide association studies (GWAS) revealed that the HAK family Na^+^-selective transporter *Zea mays L. Na^+^ content 2* (*ZmNC2*), mediating shoot Na^+^ exclusion, confers the natural variation of salt tolerance in maize [17]. Another GWAS assay found that variations in *salt-tolerance-associated-gene 4* (*SAG4*) and *SAG6* had positive roles in plant salt tolerance [18]. *ZmSOS1* and *ZmCBL8*, the important components of the SOS pathway in maize, confer natural variations in salt tolerance by regulation of Na^+^ exclusion [19].

Tomato domestication history involves two major processes dependent on fruit size: the wild *Solanum pimpinellifolium* (PIM) was domesticated in south America to *Solanum lycopersicum* var. *cerasiforme* (CER), and this process is called domestication; then CER was further improved to *Solanum lycopersicum* (BIG) in Mesoamerica, which is called improvement [20–22]. The wild tomato accommodates salt stress, while the cultivar lost tolerance during tomato domestication and improvement [23–25]. Recent progress has already identified some important variations leading to the lost salt tolerance during tomato domestication. *SlHAK20* regulates the homeostasis between Na^+^ and K^+^, and a variation in *SlHAK20* leads to the difference in Na^+^ binding ability in roots, which conferred salt tolerance variations in tomato [25]. SlDREB2 could induce the expression of *SlSOS1* against salt stress in wild tomato. Natural variations in the SlDREB2-binding *cis*-element in the promoter of *SlSOS1* down-regulates the SlDREB2-induced expression of *SlSOS1*, which increases salt sensitivity in tomato cultivars [26]. A natural variation within a *cis*-element in the promoter of the *SlSOS2* region was associated with the compromised salt tolerance during tomato domestication [27].

Due to the severe damage to plants caused by saline–alkaline stress compared with neutral salt stress, it is necessary to investigate whether saline–alkaline tolerance was selected during tomato domestication and improvement, and, if so, which variations contributed to the lost tolerance. In this study we demonstrated that saline–alkaline tolerance was compromised during tomato domestication and improvement, and a natural variation in the promoter of *SlSCaBP8* contributed to the loss of saline–alkaline tolerance during tomato improvement.

## Results

### Saline–alkaline tolerance was compromised during tomato domestication and improvement

**Figure 1:**
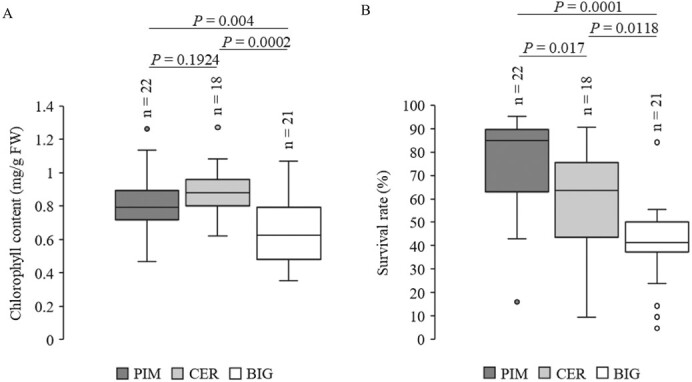
Saline–alkaline tolerance was compromised during tomato domestication and improvement. **A** Chlorophyll content of 61 tomato accessions (22 PIM, 18 CER, and 21 BIG) grown in saline–alkaline conditions (75 mM NaHCO_3_, pH 8.5) for 3 weeks. **B** Survival rates of 61 tomato accessions (22 PIM, 18 CER and 21 BIG) grown in saline–alkaline conditions (75 mM NaHCO_3_, pH 8.5) for 4 weeks and then recovered for 1 week.

To investigate whether saline–alkaline tolerance was changed during tomato domestication and improvement, we measured chlorophyll contents and survival rates of tomato accessions based on their geographical origins. Our study included 22 wild accessions of *S. pimpinellifolium* (PIM), 18 domesticated accessions of *S. lycopersicum* var. *cerasiforme* (CER), and 21 improved accessions of *S. lycopersicum* (BIG). We found that the chlorophyll contents and survival rates of the accessions in the BIG group were significantly lower compared with those in the PIM and CER groups after saline–alkaline treatments (Fig. 1, Supplementary Data Table S1). The survival rates but not chlorophyll contents showed significant differences between the PIM and CER groups. These results suggest that saline–alkaline tolerance was compromised in the process of tomato domestication and improvement.

### *SlSCaBP*8 is an important regulator of saline–alkaline tolerance in tomato

**Figure 2:**
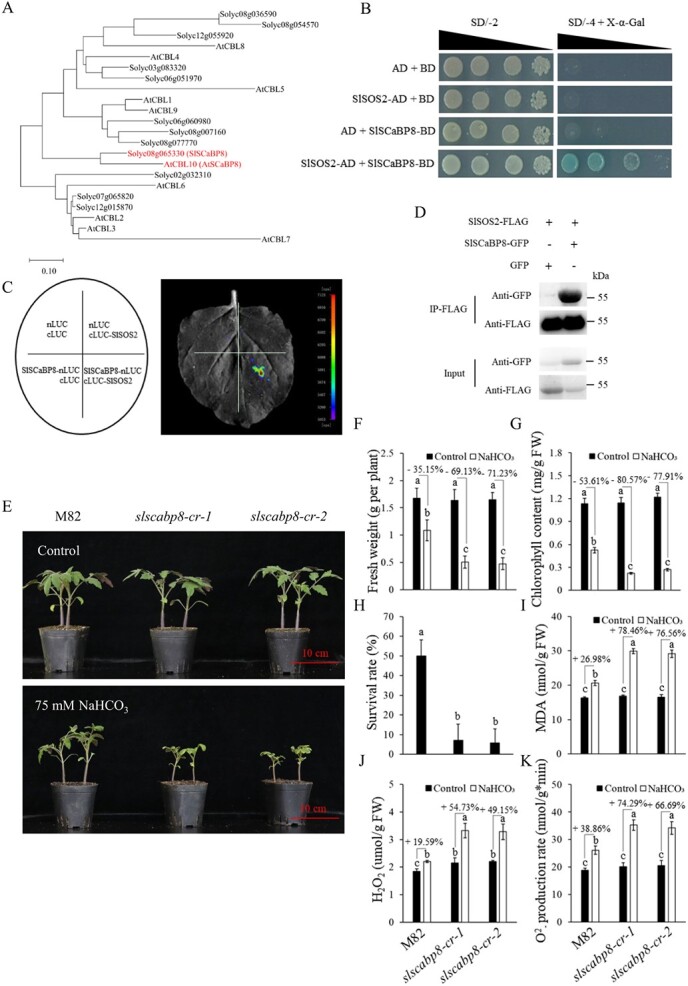
*SlSCaBP8* is a homolog of *AtSCaBP8* in tomato. **A** Phylogenetic analysis of SCaBP/CBL proteins in *Arabidopsis* and tomato. The phylogenetic tree was constructed using MEGA X [29]. The scale represents the branch length, and each node represents bootstrap values from 1000 replicates. **B** Y2H assays showing that SlSCaBP8 interacts with SlSOS2. The full-length coding sequence of *SlSCaBP8* was fused with the DNA-binding domain (BD) in pGBKT7, and the full-length coding sequence of *SlSOS2* was fused with the activation domain (AD) in pGADT7. Transformed yeast was grown on selective SD/−2 medium or SD/−4 medium plus X-α-Gal to test protein interaction. The empty pGADT7 or pGBKT7 vectors were co-transformed with SlSCaBP8-BD or SlSOS2-AD in parallel as negative controls. **C** LCI assays showing that SlSCaBP8 interacts with SlSOS2 in *N. benthamiana*. At least 12 *N. benthamiana* leaves were infiltrated and analyzed. **D** Co-IP assays showing that SlSCaBP8 interacts with SlSOS2 in *N. benthamiana. SlSOS2-FLAG* and *SlSCaBP8-GFP* vectors were co-transformed into *N. benthamiana* leaves*.***E** Phenotypes of 21-day-old M82 (wild type) and *slscabp8-cr* (*SlSCaBP8*-edited) plants grown under control and saline–alkaline conditions (75 mM NaHCO_3_, pH 8.5). Seedlings were grown in the climate chamber and maintained at 60% relative humidity under 16 h of light at 26°C and 8 h of dark at 20°C. **F**, **G** Biomass (**F**) and chlorophyll content (**G**) of M82 and *slscabp8-cr* grown in saline–alkaline conditions for 3 weeks. **H** Survival rates of M82 and *slscabp8-cr* grown in saline–alkaline conditions for 4 weeks and then recovered for 1 week. **I**–**K** MDA content (**I**), H_2_O_2_ content (**J**), and O_2_^−^ productivity rate (**K**) of M82 and *slscabp8-cr* grown under control and saline–alkaline conditions for 3 weeks. The experiments were performed with three biological replicates with similar results. Statistical significance was determined by one-way ANOVA, *P* < 0.05. Significant differences are indicated by different lowercase letters. Scale bars = 10 cm.

The EF-hand Ca^2+^ binding protein SCaBP8/CBL10 plays important roles against salt stress in shoots of *Arabidopsis* [28]. Whether *SCaBP8*/*CBL10* is involved in saline–alkaline stress in tomato has not been studied. Based on the phylogenetic tree of CBL proteins, SlSCaBP8/CBL10/Solyc08g065330 and AtSCaBP8/AtCBL10 clustered in the same clade, and this suggests that SCaBP8 is evolutionarily conserved in plants (Fig. 2A, Supplementary Data Table S2).

SCaBP8 decodes the Ca^2+^ signal and translates it to SOS2, a serine/threonine protein kinase. We performed yeast two-hybrid (Y2H) assays to confirm the interaction of SlSCaBP8 with SlSOS2. Results showed that SlSCaBP8 interacts with SlSOS2 in yeast (Fig. 2B). The interaction was further verified by the firefly luciferase complementation imaging (LCI) assay. Compared with the negative controls, a strong luminescence signal was observed in *Nicotiana benthamiana* leaves co-infiltrated with SlSCaBP8-nLUC and SlSOS2-cLUC (Fig. 2C). The co-immunoprecipitation (Co-IP) assay also supported the interaction between SlSCaBP8 and SlSOS2 (Fig. 2D). Together, these results demonstrated that SlSCaBP8 interacts with SlSOS2.

To further clarify whether *SlSCaBP8* is involved in saline–alkaline stress, we constructed the *slscabp8-cr* mutant in the cultivar M82 background by CRISPR/Cas9 gene editing technology. All the mutations in *slscabp8-cr* lead to frame shifts and generation of premature stop codons (Supplementary Data Fig. S1). As expected, *slscabp8-cr* mutants exhibited compromised tolerance of saline–alkaline stress (Fig. 2E–H). The *slscabp8-cr* mutants maintained remarkably higher malondialdehyde (MDA) contents with saline–alkaline stress than M82 (Fig. 2I), which suggested that *slscabp8-cr* mutants were subjected to more oxidative damage than M82. In line with this, significantly higher levels of H_2_O_2_ and O_2_^−^ were found in *slscabp8-cr* mutants with saline–alkaline treatments compared with that of M82 (Fig. 2J and K). It is known that the maintenance of a proper Na^+^/K^+^ ratio against saline stress in growing tissues, such as the shoot apex, is essential to maintain plant growth. To demonstrate *SlSCaBP8* function in saline–alkaline tolerance, we checked the Na^+^/K^+^ ratio in shoot apexes, adult leaves, stems, and roots. The ratio was reduced in adult leaves, stems, and roots. Na^+^ was over-accumulated in the shoot apex of *slscabp8-cr*, which caused a higher Na^+^/K^+^ ratio compared with that of M82 (Supplementary Data Fig. S2). This might be responsible for the retarded growth of *slscabp8-cr* in saline–alkaline conditions (Fig. 2E). These results suggested that *SlSCaBP8* is a positive regulator against saline–alkaline stress, and *SlSCaBP8* functions in maintaining the homeostasis of the Na^+^/K^+^ ratio.

### Pi-75 displayed enhanced saline–alkali tolerance

**Figure 3:**
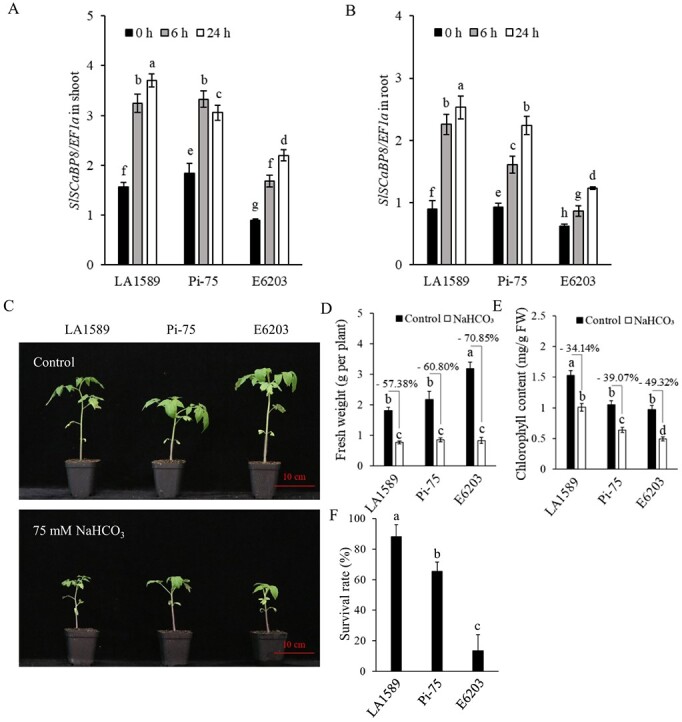
Pi-75 displayed enhanced saline–alkali tolerance. **A**, **B***SlSCaBP8* expression in LA1589, Pi-75, and E6203 in shoots (**A**) and roots (**B**) without or with NaHCO_3_ treatment. Total RNA was extracted from 5-day-old seedlings treated with 100 mM NaHCO_3_ for 0, 6, and 24 h. Tomato *EF1a* served as an internal control. **C** Phenotypes of 21-day-old LA1589, Pi-75, and E6203 grown under control and saline–alkaline conditions (75 mM NaHCO_3_, pH 8.5). Seedlings were grown in the greenhouse and maintained at 40% relative humidity under 16 h of light at 28°C and 8 h of dark at 22°C. **D**, **E** Biomass (**D**) and chlorophyll content (**E**) of LA1589, Pi-75, and E6203 grown in saline–alkaline conditions for 3 weeks. **F** Survival rates of LA1589, Pi-75, and E6203 grown in saline–alkaline conditions for 4 weeks and then recovered for 1 week. The experiments were performed with three biological replicates with similar results. Statistical significance was determined by one-way ANOVA, *P* < 0.05. Significant differences are indicated by different lowercase letters. Scale bars = 10 cm.

To clarify whether *SlSCaBP8* is selected during tomato domestication, we compared the genomic sequence of *SlSCaBP8* between LA1589 (PIM) and cultivar E6203. We found plenty of variations in the promoter regions and no variations in protein sequence, which implies that the transcription of *SlSCaBP8* in LA1589 differs from that of cultivar E6203 (Supplementary Data Fig. S3). *SlSCaBP8^LA1589^* is located in Pi-75, one of the well-established introgression-line (IL) populations derived from a cross between LA1589 and E6203 [30]. We first examined *SlSCaBP8* expression in LA1589, Pi-75, and E6203 under saline–alkaline stress. Without saline–alkaline stress, the basal level of *SlSCaBP8* expression was higher in LA1589 and Pi-75 compared with that of E6203. Saline–alkaline stress could induce *SlSCaBP8* expression in E6203, but the induction in LA1589 and Pi-75 was much stronger than that in E6203 (Fig. 3A and B). Due to the important role of *SlSCaBP8* against saline–alkaline stress, we speculate that the variations in promoter regions of *SlSCaBP8* could affect saline–alkaline tolerance. Then we compared saline–alkaline-induced shoot growth inhibition among LA1589, Pi-75, and E6203. Saline–alkaline treatment reduced the shoot fresh weight of LA1589, Pi-75, and E6203 by 57.38, 60.80, and 70.85%, respectively, compared with the mock treatment (Fig. 3C and D). LA1589 and Pi-75 also showed higher chlorophyll contents and survival rates against saline–alkaline stress compared with that of E6203 (Fig. 3E and F). Taken together, these results demonstrate that the variations in the *SlSCaBP8* promoter region affect its expression, which might lead to the saline–alkaline tolerance differences among E6203, LA1589, and Pi-75.

### *SlSCaBP8* was selected during tomato improvement

**Figure 4:**
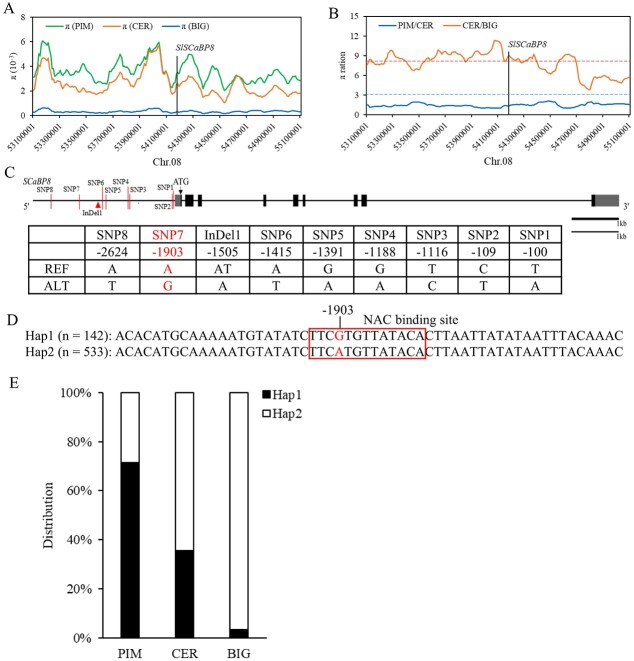
*SlSCaBP8* was selected during tomato improvement. **A** Nucleotide diversity (π) of PIM, CER, and BIG groups on chromosome 8. **B** The π ratios of PIM/CER and CER/BIG on chromosome 8. The dashed horizontal lines indicate the top 5% threshold for entire chromosome 8 (3.07 π_PIM_/π_CER_ for domestication and 8.21 π_CER_/π_BIG_ for improvement). The position of *SlSCaBP8* indicated by the vertical line is within an improvement sweep. **C** Schematic of the gene structure and positions of genetic variations in the genomic region of *SlSCaBP8* in the tomato accessions. The *SlSCaBP8* promoter is designated as 3 kb upstream of the start codon. Eight SNPs and one indel are indicated by vertical lines. REF and ALT represent the reference sequence and the alternative sequence, respectively. **D** Two haplotypes of *SlSCaBP8* in the tomato accession according to SNP7. The red nucleotide represents the nucleotide variation. **E** Distribution of Hap1 and Hap2 alleles of *SlSCaBP8* in PIM, CER, and BIG.

To further clarify whether genetic variations in *SlSCaBP8* could modulate saline–alkaline tolerance, we first analyzed the sequence variations with the resequencing data of 706 tomato accessions, which consist of 76 wild accessions from PIM, 254 domesticated accessions from CER, and 376 improved accessions from BIG (Supplementary Data Fig. S4) [31]. We calculated the nucleotide diversity (π) and the π ratios between PIM and CER and between CER and BIG on chromosome 8. We found that the π value of the *SlSCaBP8* locus was significantly lower in BIG compared with CER and PIM, and this locus was associated with an improvement rather than a domestication sweep (Fig. 4A and B, Supplementary Data Table S3). We next analyzed the SNPs (single-nucleotide polymorphisms) and InDels (insertions and deletions) in the promoter and gene body regions of *SlSCaBP8* among the 706 accessions [31]. Within 3 kb upstream of the ATG start codon of *SlScaBP8*, eight SNPs (SNP1–8) and one InDel exist (Fig. 4C, Supplementary Data Table S4). No SNP or InDel was found in the coding sequence of *SlSCaBP8.* The distributions of all these variations were remarkably reduced from PIM to CER and then to BIG (Supplementary Data Table S4). Within these variations, only SNP7 was predicted to be located in a transcription factor binding site (Fig. 4C and D).

To check whether SNP7 could lead to different transcript levels of *SlSCaBP8* in LA1589 and E6203, a transactivation assay was carried out with *proSlSCaBP8^LA1589^:LUC*, *proSlSCaBP8^LA1589^mut:LUC*, *proSlSCaBP8^E6203^:LUC*, and *proSlSCaBP8^E6203^mut:LUC*. The luminescence intensity was strongly enhanced by saline–alkaline treatment with *proSlSCaBP8^LA1589^:LUC*, and the mutation in SNP7 from G^1903^ to A^1903^ significantly reduced the luminescence signal of *proSlSCaBP8^LA1589^mut:LUC*. Saline–alkaline treatment weakly promoted the luminescence intensity with *proSlSCaBP8^E6203^:LUC*, but the mutation in SNP7 from A^1903^ to G^1903^ could significantly enhance the luminescence signal of *proSlSCaBP8^E6203^mut:LUC*. These results demonstrated that the variation SNP7 could lead to different transcription levels of *SlSCaBP8* in LA1589 and E6203 (Supplementary Data Fig. S5). SNP7 potentially impacts the expression of *SlSCaBP8* and was selected for further study*.*

**Figure 5:**
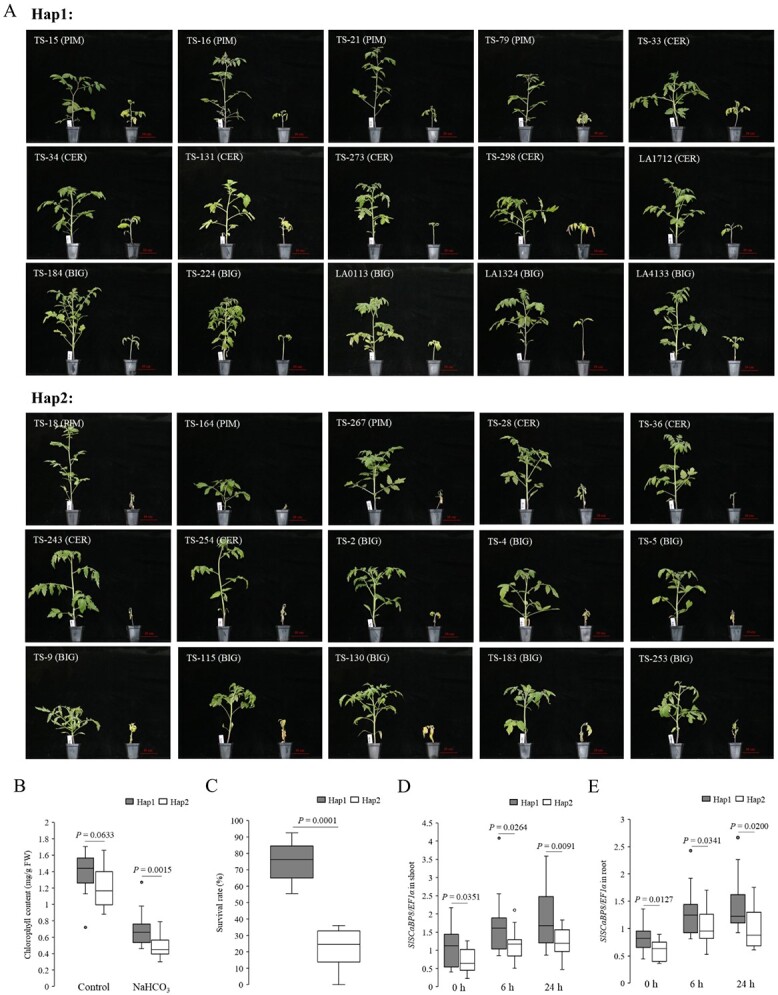
Hap1 showed enhanced saline–alkaline tolerance. **A** Phenotypes of 30 tomato accessions grown under control or saline–alkaline conditions. For the saline–alkaline condition, 30 tomato accessions were grown in saline–alkaline conditions (75 mM NaHCO_3_, pH 8.5) for 4 weeks and then recovered for 1 week. The haplotypes of *SlSCaBP8* are shown as Hap1 and Hap2, respectively. The groups of accessions are listed as PIM, CER, and BIG. Seedlings were grown in the climate chamber and maintained at 60% relative humidity under 16 h of light at 26°C and 8 h of dark at 20°C. **B** Chlorophyll content of 30 tomato accessions grown in saline–alkaline conditions for 3 weeks. The chlorophyll content was calculated with six biological replicates with similar results. **C** Survival rates of 30 tomato accessions grown in saline–alkaline conditions for 5 weeks. Survival rates were obtained from at least seven plants in three repeated experiments. **D**, **E***SlSCaBP8* expression of 30 tomato accessions in shoots (**D**) and roots (**E**).

According to the resequencing result of the *SlSCaBP8* locus, SNP7 is 1903 bp upstream of the ATG start codon. The 706 accessions were classified into two haplotypes based on SNP7 in the promoter of *SlSCaBP8*. The wild *SlSCaBP8* promoter is representative of Hap (haplotype) 1 (*n* = 142) with G^1903^, and the cultivated *SlSCaBP8* promoter belongs to Hap2 (*n* = 533) with A^1903^ (Fig. 4D, Supplementary Data Table S5). We then analyzed the phenotypes against saline–alkaline stress with the representative accessions and found that Hap1 showed higher survival rates and greater chlorophyll content compared with Hap2 (Fig. 5A–C). The transcription levels of *SlSCaBP8* were increased with saline–alkaline treatment with Hap1 varieties, and this induction was significantly reduced in Hap2 varieties (Fig. 5D and E). Consistently, the distribution of these two alleles Hap1 and Hap2 further indicated that the reduced frequency of the wild allele Hap1 is related to the compromised saline–alkaline tolerance during tomato domestication (Fig. 4E). These results show that the variation in the *SlSCaBP8* promoter region results in the compromised saline–alkaline tolerance during tomato improvement.

## Discussion

Compared with the neutral salt stress, saline–alkaline stress damages plants’ growth, development, and yield much severely. But how plants adapt to saline–alkaline stress is still not clear. Crop domestication leads to reduced stress resistance and enhanced productivity [32]. The common cultivated tomato (BIG) was domesticated from PIM and improved from CER in harsh environments in South America and Mesoamerica [20–22]. Recent progress showed that salt tolerance was lost during tomato domestication, and the core components in SOS signaling, *SlSOS1* and *SlSOS2*, were selected during this process [25–27]. Whether saline–alkaline stress tolerance was changed during tomato domestication and improvement, and what is the role of the SOS pathway, are still unknown.

In this study, we found that cultivated tomato varieties showed compromised saline–alkaline tolerance compared with their ancestors, which demonstrated that saline–alkaline tolerance was also compromised during tomato domestication and improvement. To explore the molecular mechanism against saline–alkaline stress, we focused on the EF-hand Ca^2+^ binding protein SlSCaBP8 in tomato. Y2H, LCI, and Co-IP assays proved the interaction between SlSCaBP8 and SlSOS2, and *slscabp8-cr* showed enhanced sensitivity to saline–alkaline stress. Na^+^ was over-accumulated in the shoot apex of *slscabp8-cr* and the Na^+^/K^+^ ratio was enhanced. These results suggested that *SlSCaBP8* is a positive regulator against saline–alkaline stress and that *SlSCaBP8* functions in maintaining the homeostasis of Na^+^/K^+^ ratio in tomato. We compared sequence differences of *SlSCaBP8* between PIM LA1589 and cultivar E6203 and found some potential variations in promoter regions but no variations in protein sequences, which implies that the expression of *SlSCaBP8* in LA1589 differs from that of cultivar E6203. We found that expression of *SlSCaBP8* was promoted by saline–alkaline stress, and the induction was much stronger in LA1589 and Pi-75 compared with that in E6203. Saline–alkaline tolerance of LA1589 and Pi-75 was stronger than that of E6203. These lines of data give us some cues that the potential variations in promoter regions of *SlSCaBP8* contribute to the saline–alkaline tolerance difference between LA1589 and E6203. We found eight SNPs and one InDel in the promoter region and their distribution remarkably declined from PIM to CER and then to BIG by analyzing the resequencing data, and SNP7 is located in a transcription factor binding motif. Based on SNP7, the wild *SlSCaBP8* promoter is representative of Hap1, and the cultivated *SlSCaBP8* promoter belongs to Hap2. Accessions with Hap1 showed greater saline–alkaline tolerance than that of Hap2. The distribution of Hap1 and Hap2 in PIM, CER, and BIG of the 706 accessions further suggested that the reduced frequency of the wild allele Hap1 is related to the compromised saline–alkaline tolerance during tomato domestication. The mechanism of SNP7 leading to the expression differences of *SlSCaBP8* between the wild and cultivar accessions still need to be further verified. Altogether, these results suggest that the natural variation in *SlSCaBP8* leads to weak saline–alkaline induction of *SlSCaBP8* expression and decreased saline–alkaline tolerance in the cultivated tomato, which provides a genetic variation for improving saline–alkaline tolerance in tomato.

## Materials and methods

### Plant materials and growth conditions

All tomato accessions used in this work were obtained from TGRC (Tomato Genetics Resource Center) and AGIS-CAAS (Agricultural Genomics Institute at Shenzhen, Chinese Academy of Agricultural Science). Tomato seeds were germinated at room temperature and then sown in a mixture of vermiculite and Klasmann-Deilmann substrate (1:1 v/v) and watered to saturation with water or 75 mM NaHCO_3_ (pH 8.5) solutions. In the greenhouse, seedlings were grown under 16 h of light at 28°C and 8 h of dark at 22°C, with 40% relative humidity.

### Phylogenetic tree construction

The phylogenetic tree was generated using MEGA X software [29] using the neighbor-joining method. The scale represents the branch length, and each node represents the bootstrap value from 1000 replicates. The SCaBP/CBL members in *Arabidopsis* and tomato are listed in Supplementary Data Table S2.

### Generation of mutant of *slscabp8*

Mutants of *slscabp8* were made by CRISPR/Cas9 gene editing technology as described in the previous study [33–35]. A construct containing two sgRNAs targeting *SlSCaBP8* was introduced into *Agrobacterium tumefaciens* strain LBA4404, and then transformed into tomato cultivar M82. Homozygous plants were used for further experiments. Primers are listed in Supplementary Data Table S6.

### RNA extraction and RT–qPCR

Total RNA was extracted from tomato seedlings using the TRIzol reagent (Invitrogen). cDNA was made from 2 μg of total RNA with SuperScript III reverse transcriptase (Invitrogen) and quantified on a Bio-Rad CFX96 with the SYBR Green kit (Takara). Tomato *EF1a* was used as an internal control. Statistical significance was evaluated by Student’s *t*-test. Primers are listed in Supplementary Data Table S6.

### Yeast two-hybrid assay

Yeast transformation and growth assays were performed using the Matchmaker Gold Yeast Two-Hybrid System (Clontech). *SlSCaBP8* and *SlSOS2* were cloned into pGBKT7 and pGADT7 to construct *SlSCaBP8-BD* and *SlSOS2-AD*, respectively. Both constructs were co-transformed into yeast strain Y2HGold. SD/−2 medium and SD/−4 medium plus X-α-Gal were used to check protein interaction. Empty pGADT7 or pGBKT7 vectors were co-transformed with *SlSCaBP8-BD* or *SlSOS2-AD* in parallel as negative controls. All primers used for Y2H are listed in Supplementary Data Table S6.

### Luciferase complementation imaging assay

The LCI assays were performed as described [36]. *SlSCaBP8* and *SlSOS2* were cloned into pCAMBIA1300-nLUC and pCAMBIA1300-cLUC, respectively. Both constructs were separately transformed into *A. tumefaciens* strain GV3101 for transforming *N. benthamiana* leaves. Plants were incubated at 22°C for 72 h before CCD imaging. A CCD camera (NightShade LB 985, Berthold) was used for LUC images and measurement of LUC activity. Primers used for these constructs are listed in Supplementary Data Table S6.

### Co-immunoprecipitation assays

The Co-IP assays were performed as described [37, 38]. *Agrobacterium tumefaciens strain* GV3101 carrying *p35S::SlSOS2-FLAG* or *p35S::SlSCaBP8-GFP* constructs was co-infiltrated into *N. benthamiana* leaves. Plants were incubated at 22°C for 72 h and then the co-transformed *N. benthamiana* leaves were ground to a fine powder and transferred to lysis buffer. FLAG antibody-bound agarose beads were then added to each supernatant for at least 4 h at 4°C. The precipitated samples were washed at least three times with the lysis buffer and then eluted by boiling the beads in SDS protein loading buffer for 10 min. Immunoblots were detected with anti-FLAG antibody and anti-GFP antibody. Primers used for these constructs are listed in Supplementary Data Table S6.

### Quantification of malondialdehyde content, H_2_O_2_ content and O_2_^−^ productivity rate

The MDA content, H_2_O_2_ content, and O^2−^ productivity rate were quantified according to the manufacturer’s instructions (Cominbio, catalog number MDA-1-Y for MDA, catalog number H2O2-1-Y for H_2_O_2_, and catalog number SA-1-G for O^2−^).

### Measurement of chlorophyll content

For measurement of chlorophyll content, leaf samples were incubated using a mixture of acetone and ethanol (1:1 v/v) in the dark at room temperature for 24 h. Absorbance of chlorophyll was measured at A663 and A645 [39, 40].

### Measurement of Na^+^ and K^+^ contents

Measurement of contents of Na^+^ and K^+^ of the tomato was as described in the previous study and modified [25, 41]. To determine Na^+^ and K^+^ contents, 20-day-old seedlings grown under water or 75 mM NaHCO_3_ conditions were collected. The collected plant parts were rinsed three times with double-deionized water to remove any contaminants before drying at 80°C for 24 h and then grinding to powder. Tissue powder (10 mg) was digested by 1 ml of nitric acid for 2 h in a microwave 3000 digestion system (Anton Paar). Germanium was used as the internal standard. Na^+^ and K^+^ concentrations were measured by using inductively coupled plasma mass spectrometry (ICP-MS) (ICAPQ, Thermo Fisher).

### Dual-luciferase reporter transactivation assays

For promoter activity analysis, 2.5-kb promoter sequences of *proSlSCaBP8^LA1589^*, *proSlSCaBP8^LA1589^mut*, *proSlSCaBP8^E6203^*, and *proSlSCaBP8^E6203^mut* were cloned into pGreenII 0800-LUC vector. Constructs were separately transformed into GV3101 for transforming *N. benthamiana* leaves. Plants were incubated at 22°C for 72 h and then used to detect the LUC and REN activity. Primers used for these constructs are listed in Supplementary Data Table S6.

### Data source

The variant VCF files comprising SNPs, InDels, and SVs of 706 tomato accessions were downloaded from the SolOmics database (http://solomics.agis.org.cn/tomato/ftp/) [31].

## Supplementary Material

Web_Material_uhae055

## Data Availability

The authors confirm that all data from this study are available and can be found in this article and in the supplementary information.

## References

[1] Munns R , Tester M. Mechanisms of salinity tolerance. Annu Rev Plant Biol. 2008;59:651–81 18444910 10.1146/annurev.arplant.59.032607.092911

[2] Ismail AM , Horie T. Genomics, physiology, and molecular breeding approaches for improving salt tolerance. Annu Rev Plant Biol. 2017;68:405–34 28226230 10.1146/annurev-arplant-042916-040936

[3] Ayyub CM , Ali M, Shaheen MR. et al. Enhancing the salt tolerance potential of watermelon (*Citrullus lanatus*) by exogenous application of salicylic acid. Am J Plant Sci. 2015;06:3267–71

[4] Ge Y , Li Y, Zhu YM. et al. Global transcriptome profiling of wild soybean (*Glycine soja*) roots under NaHCO3 treatment. BMC Plant Biol. 2010;10:153 20653984 10.1186/1471-2229-10-153 PMC3017823

[5] Fang S , Hou X, Liang X. Response mechanisms of plants under saline-alkali stress. Front Plant Sci. 2021;12:667458 34149764 10.3389/fpls.2021.667458 PMC8213028

[6] Zhao Y , Lu Z, He L. Effects of saline-alkaline stress on seed germination and seedling growth of *Sorghum bicolor* (L.) Moench. Appl Biochem Biotechnol. 2014;173:1680–91 24840039 10.1007/s12010-014-0956-5

[7] Guo R , Shi LX, Yan C. et al. Ionomic and metabolic responses to neutral salt or alkaline salt stresses in maize (*Zea mays* L.) seedlings. BMC Plant Biol. 2017;17:41 28187710 10.1186/s12870-017-0994-6 PMC5301417

[8] Cao Y , Zhang M, Liang X. et al. Natural variation of an EF-hand Ca^2+^-binding-protein coding gene confers saline-alkaline tolerance in maize. Nat Commun. 2020;11:186 31924762 10.1038/s41467-019-14027-y PMC6954252

[9] Sun W , Zhang H, Yang S. et al. Genetic modification of Gγ subunit AT1 enhances salt-alkali tolerance in main graminaceous crops. Natl Sci Rev. 2023;10.nwad075 10.1093/nsr/nwad075 PMC10171625 37181090

[10] Zhang H , Yu F, Xie P. et al. A Gγ protein regulates alkaline sensitivity in crops. Science. 2023;379:eade8416 36952416 10.1126/science.ade8416

[11] Yang Y , Wu Y, Ma L. et al. The Ca(2+) sensor SCaBP3/CBL7 modulates plasma membrane H(+)-ATPase activity and promotes alkali tolerance in *Arabidopsis*. Plant Cell. 2019;31:1367–84 30962395 10.1105/tpc.18.00568 PMC6588306

[12] Yang Y , Guo Y. Unraveling salt stress signaling in plants. J Integr Plant Biol. 2018;60:796–804 29905393 10.1111/jipb.12689

[13] Ma L , Ye J, Yang Y. et al. The SOS2-SCaBP8 complex generates and fine-tunes an AtANN4-dependent calcium signature under salt stress. Dev Cell. 2019;48:697–709.e5 30861376 10.1016/j.devcel.2019.02.010

[14] Mishra S , Singh B, Panda K. et al. Association of SNP haplotypes of HKT family genes with salt tolerance in Indian wild rice germplasm. Rice (N Y). 2016;9:15 27025598 10.1186/s12284-016-0083-8 PMC4811800

[15] Meyer RS , Choi JY, Sanches M. et al. Domestication history and geographical adaptation inferred from a SNP map of African rice. Nat Genet. 2016;48:1083–8 27500524 10.1038/ng.3633

[16] Campbell MT , Bandillo N, al Shiblawi FRA. et al. Allelic variants of OsHKT1;1 underlie the divergence between indica and japonica subspecies of rice (*Oryza sativa*) for root sodium content. PLoS Genet. 2017;13:e1006823 28582424 10.1371/journal.pgen.1006823 PMC5476289

[17] Zhang M , Liang X, Wang L. et al. A HAK family Na(+) transporter confers natural variation of salt tolerance in maize. Nat Plants. 2019;5:1297–308 31819228 10.1038/s41477-019-0565-y

[18] Luo X , Wang B, Gao S. et al. Genome-wide association study dissects the genetic bases of salt tolerance in maize seedlings. J Integr Plant Biol. 2019;61:658–74 30803125 10.1111/jipb.12797

[19] Zhou X , Li J, Wang Y. et al. The classical SOS pathway confers natural variation of salt tolerance in maize. New Phytol. 2022;236:479–94 35633114 10.1111/nph.18278

[20] Razifard H , Ramos A, Della Valle AL. et al. Genomic evidence for complex domestication history of the cultivated tomato in Latin America. Mol Biol Evol. 2020;37:1118–32 31912142 10.1093/molbev/msz297 PMC7086179

[21] Lin T , Zhu G, Zhang J. et al. Genomic analyses provide insights into the history of tomato breeding. Nat Genet. 2014;46:1220–6 25305757 10.1038/ng.3117

[22] Blanca J , Montero-Pau J, Sauvage C. et al. Genomic variation in tomato, from wild ancestors to contemporary breeding accessions. BMC Genomics. 2015;16:257 25880392 10.1186/s12864-015-1444-1 PMC4404671

[23] Galvez FJ . et al. Expression of LeNHX isoforms in response to salt stress in salt sensitive and salt tolerant tomato species. Plant Physiol Biochem. 2012;51:109–15 22153246 10.1016/j.plaphy.2011.10.012

[24] Pailles Y , Awlia M, Julkowska M. et al. Diverse traits contribute to salinity tolerance of wild tomato seedlings from the Galapagos Islands. Plant Physiol. 2020;182:534–46 31653717 10.1104/pp.19.00700 PMC6945843

[25] Wang Z , Hong Y, Zhu G. et al. Loss of salt tolerance during tomato domestication conferred by variation in a Na(+)/K(+) transporter. EMBO J. 2020;39:e103256 32134151 10.15252/embj.2019103256 PMC7232006

[26] Wang Z , Hong Y, Li Y. et al. Natural variations in SlSOS1 contribute to the loss of salt tolerance during tomato domestication. Plant Biotechnol J. 2021;19:20–2 32634852 10.1111/pbi.13443 PMC7769236

[27] Hong Y , Guan X, Wang X. et al. Natural variation in SlSOS2 promoter hinders salt resistance during tomato domestication. Hortic Res. 2023;10:uhac244 36643750 10.1093/hr/uhac244 PMC9832868

[28] Quan R , Lin H, Mendoza I. et al. SCABP8/CBL10, a putative calcium sensor, interacts with the protein kinase SOS2 to protect *Arabidopsis* shoots from salt stress. Plant Cell. 2007;19:1415–31 17449811 10.1105/tpc.106.042291 PMC1913747

[29] Kumar S , Stecher G, Li M. et al. MEGA X: molecular evolutionary genetics analysis across computing platforms. Mol Biol Evol. 2018;35:1547–9 29722887 10.1093/molbev/msy096 PMC5967553

[30] Doganlar S , Frary A, Ku HM. et al. Mapping quantitative trait loci in inbred backcross lines of *Lycopersicon pimpinellifolium* (LA1589). Genome. 2002;45:1189–202 12502266 10.1139/g02-091

[31] Zhou Y , Zhang Z, Bao Z. et al. Graph pangenome captures missing heritability and empowers tomato breeding. Nature. 2022;606:527–34 35676474 10.1038/s41586-022-04808-9 PMC9200638

[32] Liang Y , Liu HJ, Yan J. et al. Natural variation in crops: realized understanding, continuing promise. Annu Rev Plant Biol. 2021;72:357–85 33481630 10.1146/annurev-arplant-080720-090632

[33] Deng L , Wang H, Sun C. et al. Efficient generation of pink-fruited tomatoes using CRISPR/Cas9 system. J Genet Genomics. 2018;45:51–4 29157799 10.1016/j.jgg.2017.10.002

[34] Zhou M , Deng L, Guo S. et al. Alternative transcription and feedback regulation suggest that SlIDI1 is involved in tomato carotenoid synthesis in a complex way. Hortic Res. 2022;9:uhab045 10.1093/hr/uhab045 PMC8788357 35031800

[35] Yang T , Ali M, Lin L. et al. Recoloring tomato fruit by CRISPR/Cas9-mediated multiplex gene editing. Hortic Res. 2023;10:uhac214 36643741 10.1093/hr/uhac214 PMC9832834

[36] Chen H , Zou Y, Shang Y. et al. Firefly luciferase complementation imaging assay for protein-protein interactions in plants. Plant Physiol. 2008;146:368–76 18065554 10.1104/pp.107.111740 PMC2245818

[37] Liu L , Zhang Y, Tang S. et al. An efficient system to detect protein ubiquitination by agroinfiltration in *Nicotiana benthamiana*. Plant J. 2010;61:893–903 20015064 10.1111/j.1365-313X.2009.04109.x

[38] Zhang X , Zhou W, Chen Q. et al. Mediator subunit MED31 is required for radial patterning of *Arabidopsis* roots. Proc Natl Acad Sci USA. 2018;115:E5624–e5633 29844159 10.1073/pnas.1800592115 PMC6004453

[39] Arnon DI . Copper enzymes in isolated chloroplasts. POLYPHENOLOXIDASE in *Beta vulgaris*. Plant Physiol. 1949;24:1–15 16654194 10.1104/pp.24.1.1 PMC437905

[40] Li L , Li Q, Chen B. et al. Identification of candidate genes that regulate the trade-off between seedling cold tolerance and fruit quality in melon (*Cucumis melo* L.). Hortic Res. 2023;10:uhad093 10.1093/hr/uhad093 PMC10321389 37416729

[41] Geng S , Sohail H, Cao H. et al. An efficient root transformation system for CRISPR/Cas9-based analyses of shoot–root communication in cucurbit crops. Hortic Res. 2022;9:uhab082 10.1093/hr/uhab082 PMC9071382 35048110

