# Supplementary material for: A natural variation in SlSCaBP8 promoter contributes to the loss of saline–alkaline tolerance during tomato improvement
Source: Hortic Res. 2024 Feb 23;11(4):uhae055. doi: 10.1093/hr/uhae055 (PMC11040208; doi:10.1093/hr/uhae055)
Supplement: Web_Material_uhae055 [file web_material_uhae055.zip › 20240118 Suppemental figures.pptx]

## Slide 1
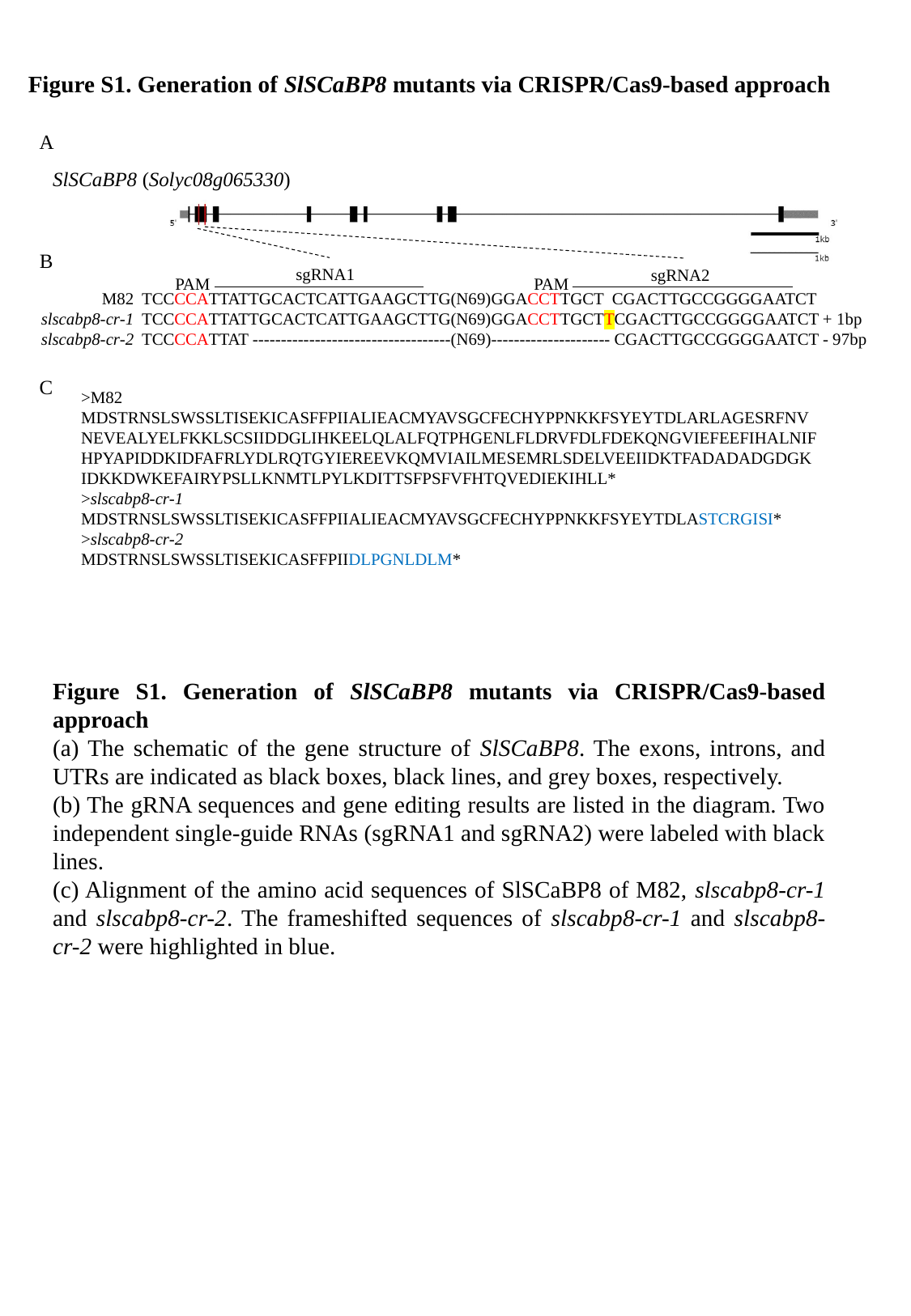

Figure S1. Generation of SlSCaBP8 mutants via CRISPR/Cas9-based approach
A
SlSCaBP8 (Solyc08g065330)
sgRNA1
sgRNA2
PAM
PAM
M82
slscabp8-cr-1
slscabp8-cr-2
TCCCCATTATTGCACTCATTGAAGCTTG(N69)GGACCTTGCT CGACTTGCCGGGGAATCT
TCCCCATTATTGCACTCATTGAAGCTTG(N69)GGACCTTGCTTCGACTTGCCGGGGAATCT + 1bp
TCCCCATTAT -----------------------------------(N69)--------------------- CGACTTGCCGGGGAATCT - 97bp
>M82
MDSTRNSLSWSSLTISEKICASFFPIIALIEACMYAVSGCFECHYPPNKKFSYEYTDLARLAGESRFNVNEVEALYELFKKLSCSIIDDGLIHKEELQLALFQTPHGENLFLDRVFDLFDEKQNGVIEFEEFIHALNIFHPYAPIDDKIDFAFRLYDLRQTGYIEREEVKQMVIAILMESEMRLSDELVEEIIDKTFADADADGDGKIDKKDWKEFAIRYPSLLKNMTLPYLKDITTSFPSFVFHTQVEDIEKIHLL*
>slscabp8-cr-1
MDSTRNSLSWSSLTISEKICASFFPIIALIEACMYAVSGCFECHYPPNKKFSYEYTDLASTCRGISI*
>slscabp8-cr-2
MDSTRNSLSWSSLTISEKICASFFPIIDLPGNLDLM*
B
C
Figure S1. Generation of SlSCaBP8 mutants via CRISPR/Cas9-based approach
(a) The schematic of the gene structure of SlSCaBP8. The exons, introns, and UTRs are indicated as black boxes, black lines, and grey boxes, respectively.
(b) The gRNA sequences and gene editing results are listed in the diagram. Two independent single-guide RNAs (sgRNA1 and sgRNA2) were labeled with black lines.
(c) Alignment of the amino acid sequences of SlSCaBP8 of M82, slscabp8-cr-1 and slscabp8-cr-2. The frameshifted sequences of slscabp8-cr-1 and slscabp8-cr-2 were highlighted in blue.

## Slide 2
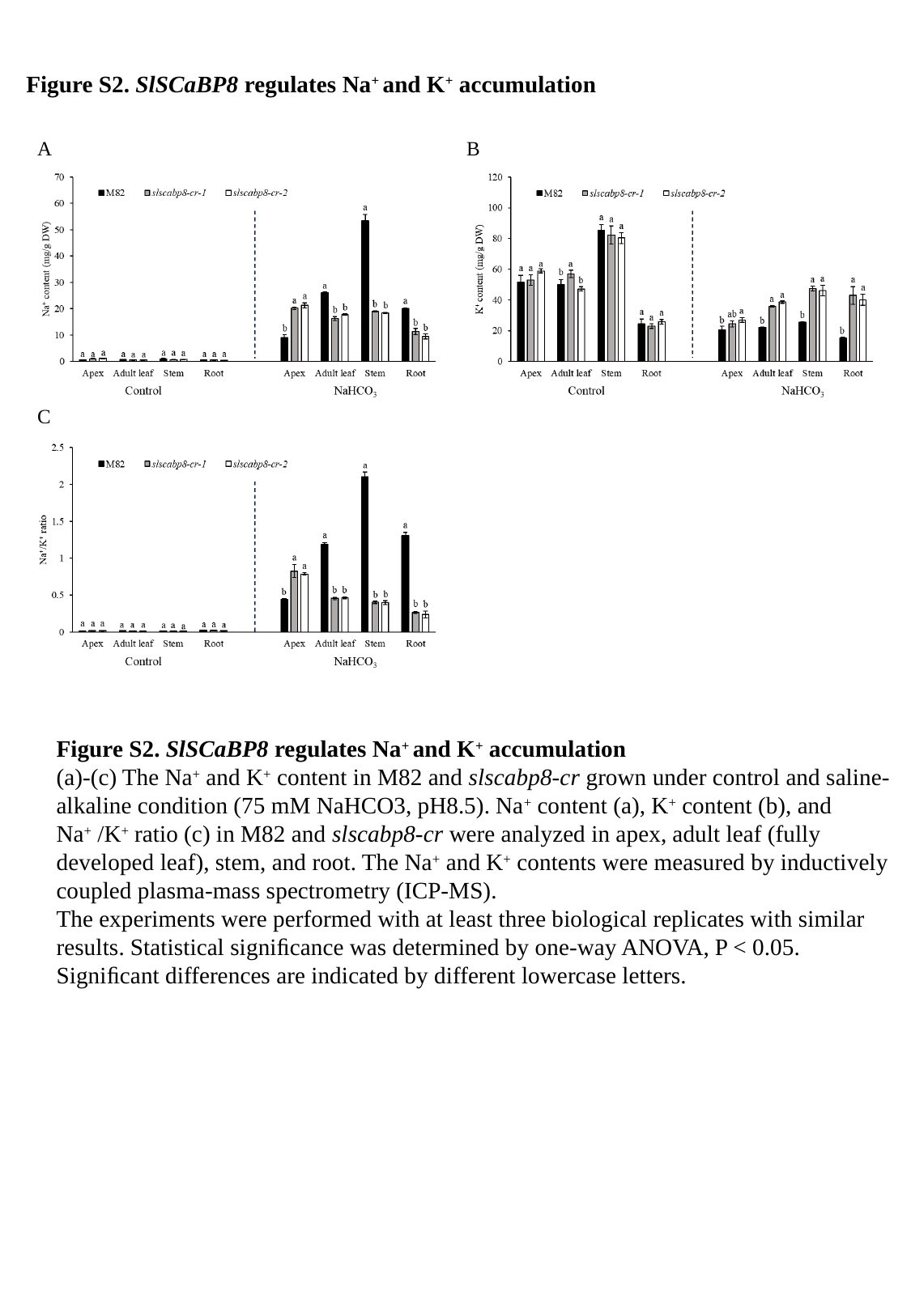

Figure S2. SlSCaBP8 regulates Na+ and K+ accumulation
A
B
C
Figure S2. SlSCaBP8 regulates Na+ and K+ accumulation
(a)-(c) The Na+ and K+ content in M82 and slscabp8-cr grown under control and saline-alkaline condition (75 mM NaHCO3, pH8.5). Na+ content (a), K+ content (b), and Na+ /K+ ratio (c) in M82 and slscabp8-cr were analyzed in apex, adult leaf (fully developed leaf), stem, and root. The Na+ and K+ contents were measured by inductively coupled plasma-mass spectrometry (ICP-MS).
The experiments were performed with at least three biological replicates with similar results. Statistical signiﬁcance was determined by one-way ANOVA, P < 0.05. Signiﬁcant differences are indicated by different lowercase letters.

## Slide 3
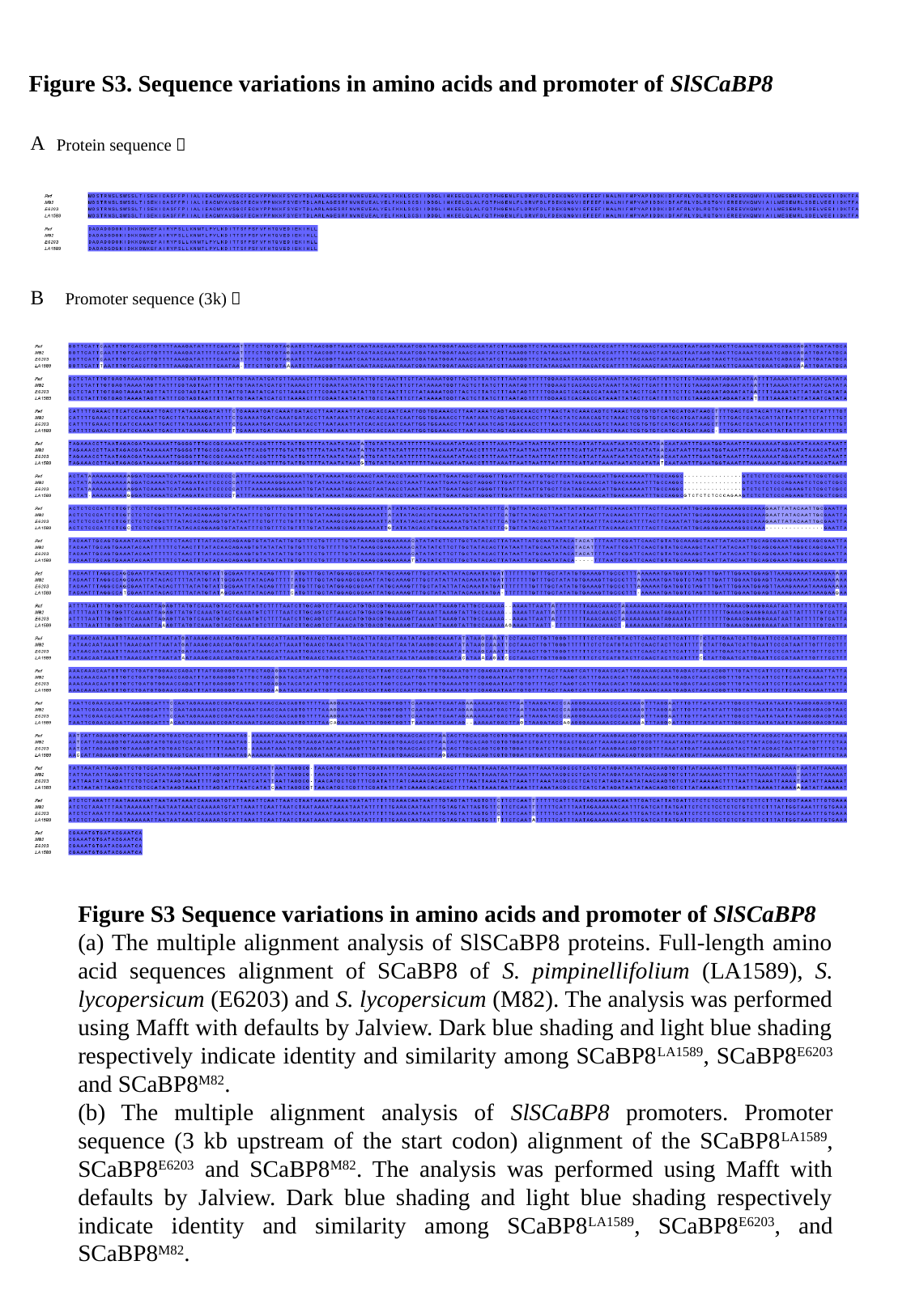

Figure S3. Sequence variations in amino acids and promoter of SlSCaBP8
A
Protein sequence：
B
Promoter sequence (3k)：
Figure S3 Sequence variations in amino acids and promoter of SlSCaBP8
(a) The multiple alignment analysis of SlSCaBP8 proteins. Full-length amino acid sequences alignment of SCaBP8 of S. pimpinellifolium (LA1589), S. lycopersicum (E6203) and S. lycopersicum (M82). The analysis was performed using Mafft with defaults by Jalview. Dark blue shading and light blue shading respectively indicate identity and similarity among SCaBP8LA1589, SCaBP8E6203 and SCaBP8M82.
(b) The multiple alignment analysis of SlSCaBP8 promoters. Promoter sequence (3 kb upstream of the start codon) alignment of the SCaBP8LA1589, SCaBP8E6203 and SCaBP8M82. The analysis was performed using Mafft with defaults by Jalview. Dark blue shading and light blue shading respectively indicate identity and similarity among SCaBP8LA1589, SCaBP8E6203, and SCaBP8M82.

## Slide 4
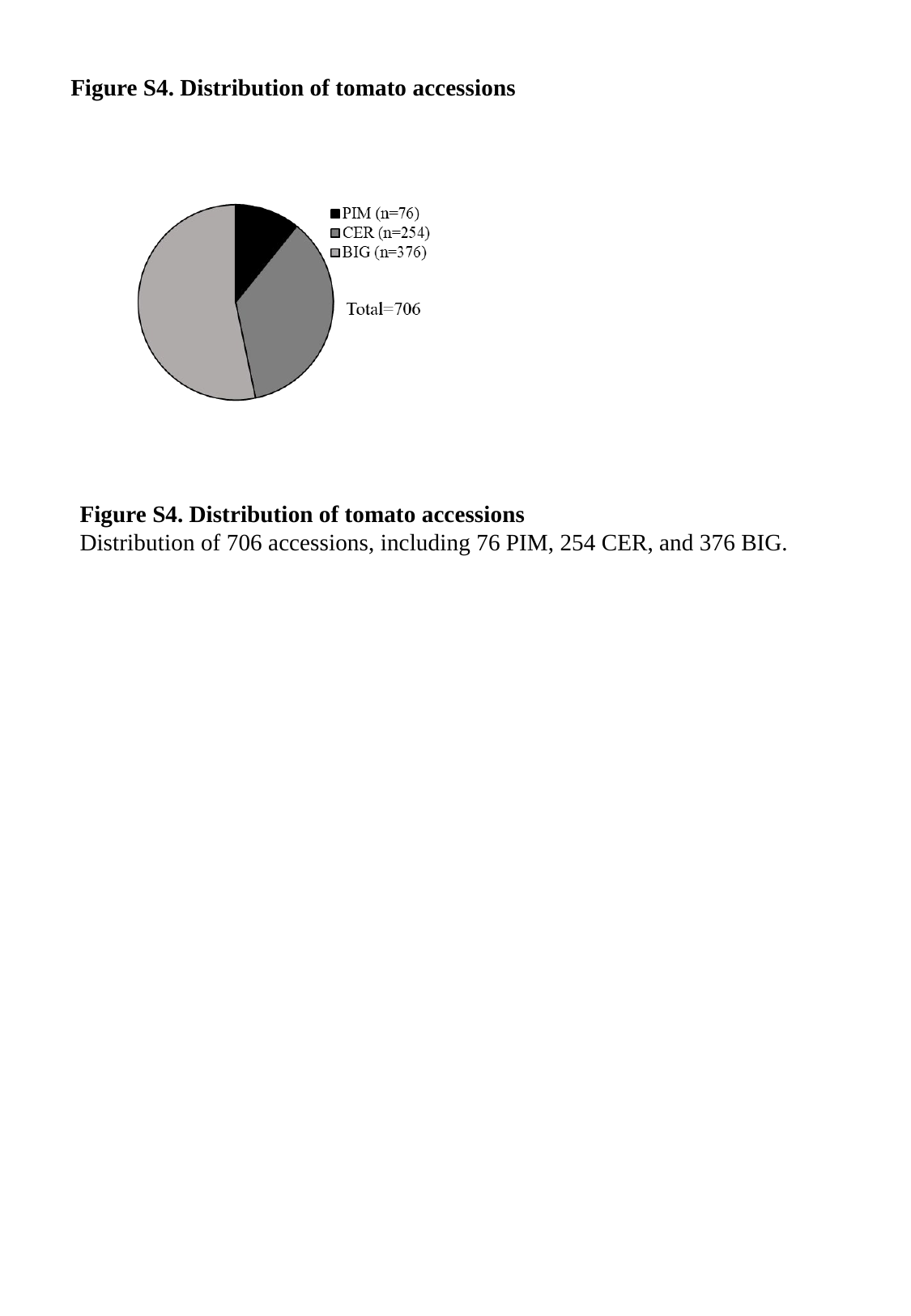

Figure S4. Distribution of tomato accessions
Figure S4. Distribution of tomato accessions
Distribution of 706 accessions, including 76 PIM, 254 CER, and 376 BIG.

## Slide 5
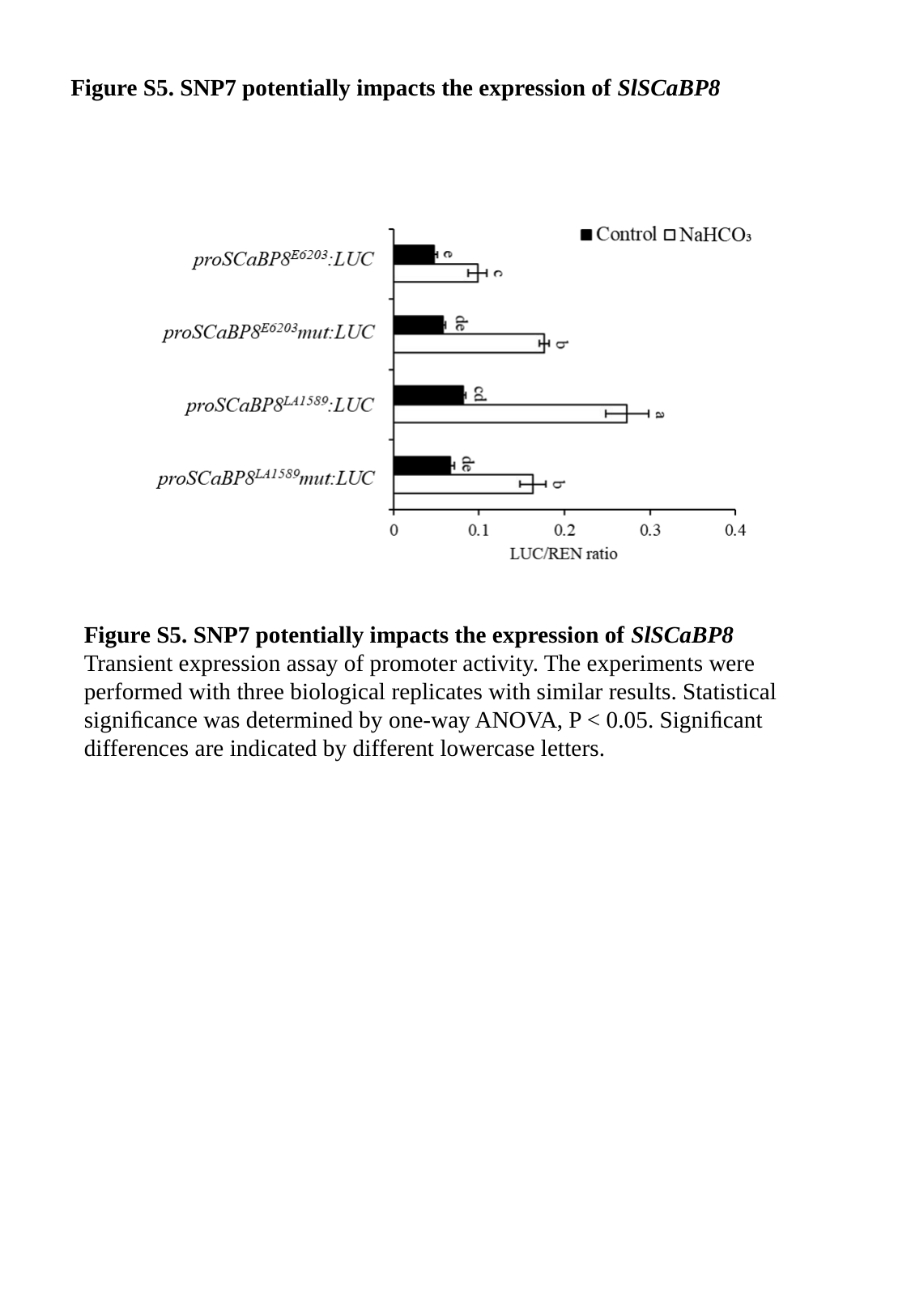

Figure S5. SNP7 potentially impacts the expression of SlSCaBP8
Figure S5. SNP7 potentially impacts the expression of SlSCaBP8
Transient expression assay of promoter activity. The experiments were performed with three biological replicates with similar results. Statistical signiﬁcance was determined by one-way ANOVA, P < 0.05. Signiﬁcant differences are indicated by different lowercase letters.
